# Molecular Evolution of Plant SULTR Proteins and Expression Analysis of HvSULTR Under Heat Stress in Barley

**DOI:** 10.3390/plants14203165

**Published:** 2025-10-15

**Authors:** Chunmeng Zhu, Xuan Chen, Li Hao, Wessam A. Abdelrady, Tao Tong, Fenglin Deng, Fanrong Zeng, Zhong-Hua Chen, Xiaojian Wu, Wei Jiang

**Affiliations:** 1 MARA Key Laboratory of Sustainable Crop Production in the Middle Reaches of the Yangtze River (Co-Construction by Ministry and Province), Hubei Key Laboratory of Waterlogging Disaster and Agricultural Use of Wetland, College of Agriculture, Yangtze University, Jingzhou 434025, China; chunmeng-zhu.stu@yangtzeu.edu.cn (C.Z.); chenxuan.stu@yangtzeu.edu.cn (X.C.);; 2 Zhejiang Key Laboratory of Crop Germplasm Resource, Department of Agronomy, College of Agriculture and Biotechnology, Zhejiang University, Hangzhou 310058, China; 3 China National Rice Research Institute, Hangzhou 311401, China; 4 School of Agriculture, Food and Wine, Waite Research Institute, The University of Adelaide, Glen Osmond, SA 5064, Australia; 5 Xianghu Laboratory, Hangzhou 311231, China; 6 College of Agricultural, Nanjing Agricultural University, Nanjing 210095, China

**Keywords:** evolution, expression analysis, sulfate transporters, *Hordeum vulgare* L., transcriptome, gene family, abiotic stress

## Abstract

Sulfur metabolism plays an important role in plant growth and environmental adaptation. Sulfate transporters (*SULTRs*) are essential players that mediate sulfur acquisition and distribution in many plants, thereby influencing the cellular redox homeostasis under abiotic stress. In this study, we identified 16 putative *HvSULTRs* genes in barley at the genome-wide level. The conservation and divergence of the *SULTR* gene family were assessed through a phylogenetic tree and gene structure analysis, revealing that these genes are closely distributed along the chromosomes. Furthermore, the expression pattern of *SULTRs* in multiple tissues, including flower, root, leaf, stem, seeds, female, male, root meristem, and apical meristem, were analyzed among ten land plants using a public database. Interestingly, the expression of *HvSULTR2, HvSULTR4*, and *HvSULTR5* was upregulated after four days of heat treatment, suggesting their importance in barley’s adaptive response to heat stress. In addition, *HvSULTR11* was confirmed to be localized at the plasma membrane and display functional interactions with Hv14-3-3A/Hv14-3-3D. In addition, haplotypes of the *HvSULTR11* based on SNP (Single Nucleotide Polymorphism) were divided into ten types across 123 barley varieties. Together, these results provide a new clue to clarify the molecular mechanism of *SULTRs* in stress response and a new candidate gene resource to enhance the stress (e.g., heat and drought) tolerance in barley.

## 1. Introduction

Sulfur (S) is an indispensable nutrient for plant growth, ranking fourth in content among the essential elements. It supports plant growth and development, enhances resistance to biotic (e.g., pests) and abiotic (e.g., heat, drought, and salinity) stresses, and affects crop yield and quality [1]. It is also a structural component of various prosthetic groups such as coenzymes, iron–sulfur centers, lipoic acid, and thiamine. Sulfur participates in numerous metabolic processes, including the production of amino acids like cysteine and methionine, which can regulate protein folding or activity [2]. Meanwhile, it is also an indispensable key factor in the synthesis of glutathione, which is important to maintain the dynamic balance of cells while alleviating the damage caused by oxidative stress [3]. Besides, the key pathway of S metabolism, chloroplast-to-nucleus SAL1–PAP retrograde signaling coordinates photosynthetic and extracellular reactive oxygen species (ROS) production with abscisic acid (ABA) signaling, providing a central integration node for stress responses. This pathway is evolutionarily conserved and has been linked to the emergence of land-plant drought responses via guard-cell regulation. In soil, S generally exists in the form of sulfate (SO_4_^2−^), which is categorized as a major inorganic ion to plant cell [4]. Plants absorb it through their root systems, then transport it to various above-ground tissues, and finally assimilate and utilize it [5,6].

In plants, the transmembrane transport of SO_4_^2−^ is mainly mediated by sulfate transporters (SULTRs), which play the critical role in the absorption of SO_4_^2−^ by plant roots [7], as well as in the long-distance transport and distribution of SO_4_^2−^ within plants [8,9]. SULTRs have been identified in many plant species, including *Arabidopsis thaliana* [10], wheat [11], rice [12], *Brassica oleracea* [13], cotton [14], blueberry [15], *Triticum turgidum* [16], *Brassica napus* [17], common bean [18], etc. SULTR is encoded by multiple genes, with 10–12 transmembrane domains, and its C-terminal region contains a Sulphate Transporter and Anti-Sigma factor antagonist (STAS) domain that regulates the activity, synthesis, and stability of SULTR [19]. In our previous study, SULTRs were found in the Rhodophyta *Cyanidioschyzon merolae*, *Chlorophyta Ostreococcus sp*. and *Volvox carteri*, *Streptophyta Klebsormidium flaccidum*, and all land plants examined, indicating a conserved function in plants [20]. Furthermore, SULTRs also participate in abiotic stresses, as sulfate might improve the ABA synthesis in guard cells, which contain a chloroplastic *SULTR3;1* transporter [21]. In addition, *OsSultr1;1* can also enhance the tolerance of *A. thaliana* to heavy metal stress [22].

Heat stress adversely affects plant growth and development worldwide [23,24,25,26]. Plants has evolved a number of physiological traits to adapt to the fluctuating environment in the process of territorialization [27]. Plants normally cope with heat and drought stress by modulating their stomatal conductance, photosynthetic activity, cellular oxidative conditions, metabolomic profiles, and molecular signaling mechanisms [28]. Notably, sulfur assimilation must be rebalanced during drought, requiring coordination between the primary and secondary sulfur metabolism, referred to as the sulfur assimilation conundrum, with consequences for thiol pools and stress tolerance. For instance, at the cellular level, drought signals can promote the production of osmotic substances like proline and trehalose, activate the antioxidant system to maintain redox homeostasis, and also trigger responses through plant hormone pathways including ABA, brassinosterol (BR), and ethylene (ET) [23,24,29,30,31]. Furthermore, numerous genes, such as MID1-Complementing Activity 1 (MCA1), MCA2 [32], Calcium Permeable Stress-Gated Cation Channel 1 (CSC1) [33], Ca^2+^ Increase1 (OSCA1) [34], and Dehydration-Responsive Element Binding Protein 2a (DREB2A) [35], have been identified as critical factors for drought tolerance. Heat stress affects the stability of protein, membrane, RNA types, and cytoskeletal structure, and alters the efficiency of enzymatic reactions in cells, leading to metabolic imbalance [36]. Thus, plants have formed complex and efficient regulatory networks to resist and adapt to it. It has been reported that cyclic nucleotide-gated calcium channel 14 (CNGC14), CNGC16 in rice [37], and CNGC2, CNGC4, CNGC6 in *A*. *thaliana* [38,39] are involved in the heat-shock-dependent flow of Ca^2+^ into the cytoplasm. Additionally, heat-shock transcription factors (HSFs) play important roles in plant responses to heat stress by regulating the expression of stress-responsive genes [40,41]. In barley (*Hordeum vulgare* L.), a series of proteins related to ROS, ion transport, stomatal movement, and hormone signaling have been reported to be associated with drought and heat tolerance, for instance Jasmonate ZIM-domain 2 (HvJAZ2) [42], Zinc-Induced Facilitator-Like 2 (HvZIFL2), and Peroxidase 11 (HvPOD11) [43], PHOTOPERIOD 1 (HvPPD-H1) [44], MADS-box 1 (HvMADS1) [45], and so on. Recently, the SUTLR proteins have been proved to respond to biotic and abiotic stresses in plants, such as salt stress [16], phosphorus [46], chromate [47], sulfur [48], selenium [49], and diseases [13]. However, the role of SULTR under heat and drought remains unknown.

Barley is the fourth cereal crop in the world in terms of total production area and yield [50]. Up to the present, many genes have been proved to regulate the tolerance to heat and drought in barley [41,51,52,53,54]. The SULTR-like phosphorus distribution transporter (HvSPDT) is node-localized and required for distributing phosphate to barley grains [55]. However, the functional characterization and associated features of the *SULTR* gene family are still unknown under heat and drought stress in barley. In this study, bioinformatics tools were used to analyze the genomic structure, domain organization, and the expansion of SULTR gene family. Furthermore, expression profiles of those genes were analyzed in different barley organs using the publicly available resources. In addition, we found the interaction between HvSULTR11 and Hv14-3-3A/Hv14-3-3D. Thus, we proposed that SULTR genes play an important role in heat response through regulating photosynthesis in barley.

## 2. Results

### 2.1. Evolution and Expression Profiles of SULTRs in Plants

**Figure 1:**
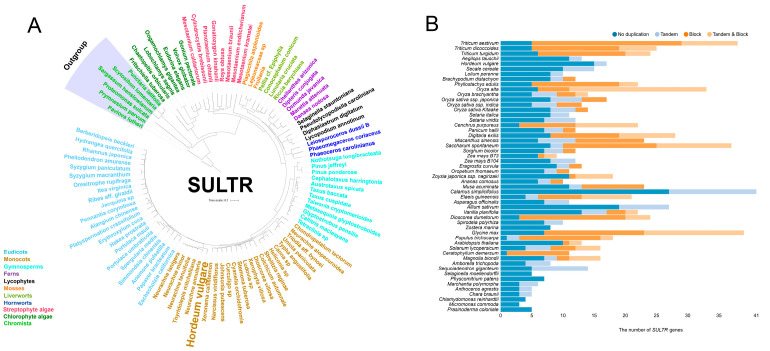
Phylogenetic tree analysis of SULTR in green plants (**A**). All sequences were obtained from the OneKP and Phytozome website. Tandem and block gene duplication of the SULTR gene family in *Chlorophyta* and *Embryophyta* (**B**). All of the gene numbers were downloaded from the PLAZA database containing >100 plant and algal species.

To explore the evolutionary relationships within the *SULTR* family, a phylogenetic tree was constructed using the neighbor-joining (NJ) method based on multiple sequence alignments of SULTR sequences from representative plant species obtained from the public databases, indicating that SULTR might evolve from *Chromista* (Figure 1A). In the 53 investigated plant species, a total of 859 *SULTR* family members were detected. The amount of *SULTR* genes varied across plant species, with the angiosperms having about 3–8 times of *SULTR* genes than algae (Figure 1B). Furthermore, 15%, 27%, and 15% of the *SULTR* genes exhibited tandem, block, and duplication, respectively (https://bioinformatics.psb.ugent.be/plaza/versions/plaza_v5_monocots/gene_families/view/HOM05M000293 accessed on 4 July 2025). These results indicate that the *SULTR* family underwent a potential evolutionary expansion, which led to the evolutionary divergence and functional differentiation of *SULTR* genes in green plants.

**Figure 2:**
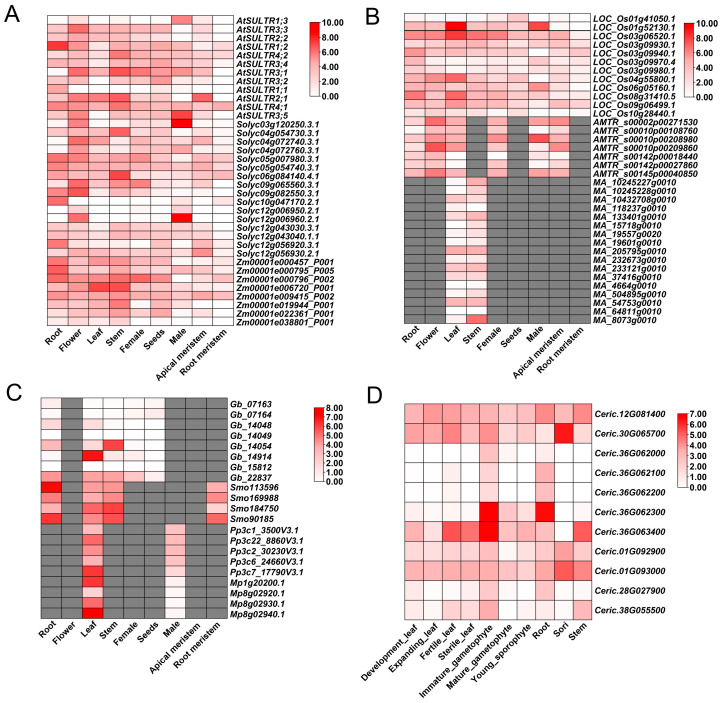
Comparative expression of SULTR genes across tissues and plant lineages. (**A**) Eudicot and maize panel: *Arabidopsis thaliana* (*At*), *Solanum lycopersicum* (*Solyc*), and *Zea mays* (*Zm*). (**B**) Monocot and basal angiosperm panel: *Oryza sativa* (LOC_Os), *Musa acuminata* (*MA*), and *Amborella trichopoda* (*AMTR*). (**C**) Gymnosperm and early-diverging land plants: *Ginkgo biloba* (*Gb*), lycophyte *Selaginella moellendorffii* (*Smo*), moss *Physcomitrium patens* (*Pp*), and liverwort *Marchantia polymorpha* (*Mp*). (**D**) Fern: *Ceratopteris richardii* (*Ceric*). Heatmaps show expression (log-scaled) across male, female, apical meristem, root meristem, flower, seed, root, leaf, and stem (or the closest available homologous tissues per species). White implying low expression while red implying high expression, and missing data set as gray.

We then used CoNekT to compare the expression profiles of *SULTR* orthologues in multiple plant species (https://evorepro.sbs.ntu.edu.sg/heatmap/comparative/family/396/raw, accessed on 21 May 2025; Figure 2 and Figure S2). *SULTR* family members are expressed in every major land-plant clade, yet their strongest and most consistent enrichment occurs in roots (Figure 2). For instance, *AtSULTR4;1* and *AtSULTR3;5* in *A. thaliana, Solyc06g084140.4.1*, and *Solyc04g072760.3.1* in *Solanum lycopersicum*, *Zm00001e006720_P001*, and *Zm00001e009415_P002* in *Zea mays* show pronounced root expression (Figure 2A). Similarly, *LOC_Os03g09940.1*, *LOC_Os03g09970.4*, and *LOC_Os03g09980.1* in *Oryza sativa*, *AMTR_s00145p00040850* in *Amborella trichopoda*, and *MA_19557g0020*, *MA_54753g0010*, and *MA_64811g0010* in *Musa acuminata* are root-enriched (Figure 2B). Outside angiosperms, *Smo184750* in *Selaginella moellendorffii*, and *Pp3c1_3500V3.1* in *Physcomitrium patens* exhibited much higher expression in root or root-like tissues (Figure 2C and Figure S1), and several *Ceratopteris richardii* homologues display strong expression in developing leaves and gametophytes (Figure 2D). Furthermore, many *SULTR* genes also showed strong expression in plant aerial parts, such as *Mp.54682.g010*, *Glyma17g20520.1*, *Mp8g02920.1–Mp8g02940.1*, etc. (Figure 2 and Figure S1). These findings highlight the critical role of these *SULTR* genes in sulfate acquisition from the soil and translocation to aerial parts of land plants. In addition, *SULTR* genes were also found to be preferentially expressed in floral organs, such as *Smo169988* in lycophyte *S. moellendorffii*, *Gb_14054* in gymnosperm *Ginkgo biloba*, and *LOC_Os01g52130.1* in rice, indicating their additional specialized function in the development of reproductive organs (e.g., rice male tissue, ginkgo cones) (Figure 2B,C).

It was notable that some *SULTR* genes, such as *At1g77990*, *At4g26560*, and *Zm00001d020462*, demonstrated the upregulated expression under nutrient limitation and abiotic stress conditions (Figure 2). These stress-responsive genes tended to cluster in the same phylogenetic tree (Figure S1), suggesting that these *SULTR* genes may play the common roles in plants’ adaptive responses to environmental challenges and share similar regulatory mechanisms. Furthermore, some stress-responsive genes like *At1g77990* were enriched with stress-responsive motifs, which confirms the alignment of expression profiles with structural features, including conserved motifs and domains.

Taken together, the present comprehensive analysis provides a detailed overview of *SULTR* gene expression across species, tissues, and conditions. The results highlight these genes’ structural and functional diversity, underscoring their specialized roles in sulfate uptake, transport, and environmental adaptation. These findings offer valuable insights into the evolution of *SULTR* genes and their potential applications in improving crop nutrient efficiency.

### 2.2. Identification and Gene Structure Comparison of HvSULTRs in Barley

**Figure 3:**
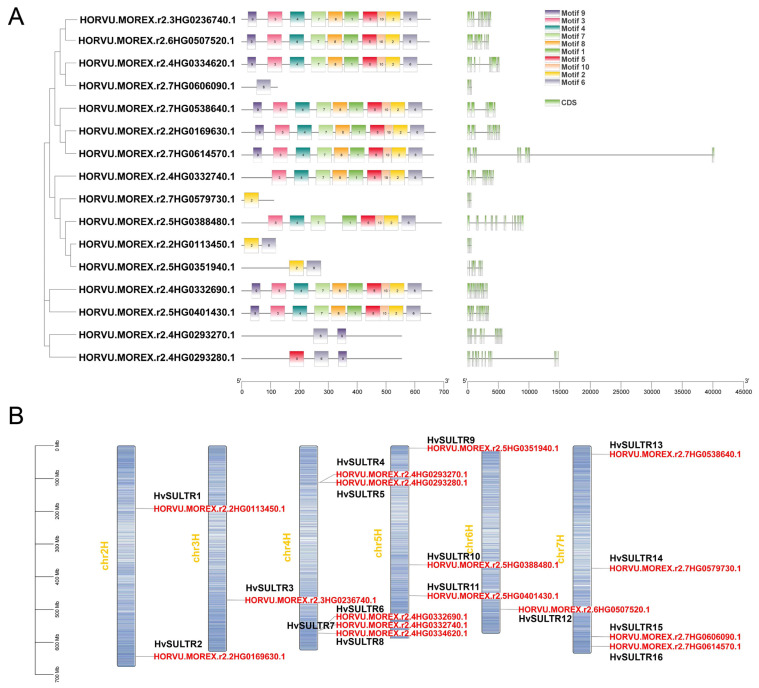
Gene structure and motif (**A**) and chromosomal location (**B**) of HvSULTRs. Genes were named according to the order of chromosome location, e.g., HORVU.MOREX.r2.2HG0113450.1 named HvSULTR1. The figures were visualized using Tbtools (Tbtools.v2.056).

A comprehensive analysis of the gene structures and motifs among the *HvSULTR* genes was conducted to compare the HvSULTR at both the protein and nucleic acid levels. We examined the exon–intron arrangements to investigate the structural diversity within the *HvSULTR* gene family. We found a considerable variation in the number and length of exons and introns across *HvSULTR* genes (Figure 3A), suggesting the potential functional differentiation within the *HvSULTR* family. The motif patterns within the *HvSULTR* family were analyzed to further explore the structural diversity and functional properties of *HvSULTR* proteins. Motifs 1–10, identified using the MEME search tool, represent conserved regions of functional importance. Motif 3 is a relatively conserved sequence (Figure S2). The distribution and composition of these motifs varied among *HvSULTR* members, indicating structural and functional diversity. While several HvSULTR proteins shared similar motif compositions, others showed distinct motif arrangements, reflecting potential specialization in their functional roles. Additionally, the conserved domains of HvSULTR proteins were analyzed, confirming that all *HvSULTR* proteins contained the SULTR domain. These results corroborate the phylogenetic analysis and support the subfamily classification of *HvSULTR* genes. Based on the distribution of conserved domains and motifs and previous studies on *SULTR* gene families in plants, the phylogenetic tree was classified into distinct groups. The predicted *HvSULTR* genes were categorized into several groups representing different evolutionary lineages. Together, the structural diversity in exon–intron organization and motif distribution highlights the evolutionary divergence and functional specialization of the *HvSULTR* gene family.

The chromosomal distribution of *HvSULTR* genes in barley revealed their localization across seven chromosomes (from chr2H to chr7H), highlighting both clustered and dispersed arrangements (Figure 3B). We named the genes based on the chromosomal location distribution of *HvSULTR* genes (Table S2). On chr2H, two genes, *HvSULTR1* and *HvSULTR2*, were identified, while Chr3H harbored a single gene, *HvSULTR3*, indicating their involvement in diverse functions. Chr4H exhibited a notable cluster of five *HvSULTR* genes, from *HvSULTR4* to *HvSULTR8*. This clustering suggests possible gene duplication events and functional specialization. Chr5H, Chr6H, and Chr7H contained three, one, and four genes, respectively, indicating their likely involvement in stress or developmental processes. These findings may provide insights into the regulatory mechanisms and functional diversity of *HvSULTR* genes in barley.

### 2.3. Expression Profiles of HvSULTR Genes Across Multiple Tissues and upon Abiotic Stresses

**Figure 4:**
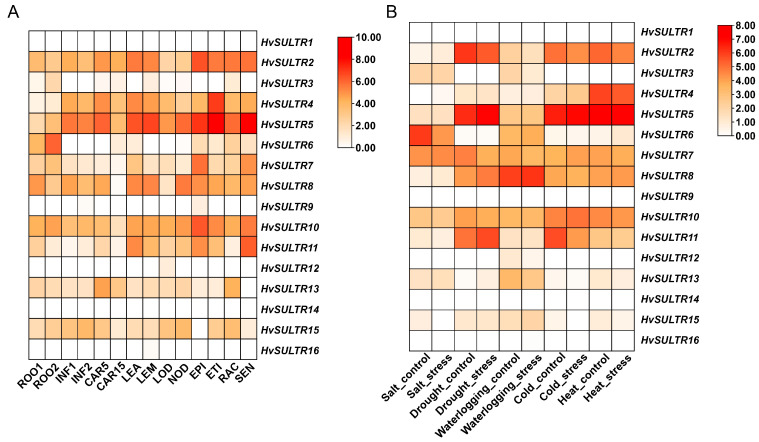
Gene expression analysis of HvSULTR in diverse tissues (**A**) and stress conditions (**B**). Data were downloaded from a public database (https://apex.ipk-gatersleben.de/apex/f?p=284:10 and http://barleyexp.com/, accessed on 15 March 2025). White implying low expression and red implying high expression.

The expression analysis of *HvSULTR* genes across various tissues and under different abiotic stress conditions revealed a complex regulatory network, highlighting their functional diversity and specialization (Figure 4A). For example, *HvSULTR8* exhibited significant expression in leaf tissues (LEA, 36.25) and early inflorescence (INF1, 23.22), indicating its critical role in supporting sulfate transport during vegetative and reproductive phases. Similarly, *HvSULTR6* was predominantly expressed in roots (12.15), senescing tissues (SEN, 24.60), and nodules (NOD, 11.60), suggesting its involvement in primary sulfate uptake and redistribution during nutrient remobilization. Root-specific expression was evident for genes like *HvSULTR2*, which showed strong expression in ROOT (47.62) and CAR5 (59.26), emphasizing their importance in sulfate acquisition and transport to reproductive tissues. Conversely, genes such as *HvSULTR14* and *HvSULTR1* exhibited negligible expression across all tissues, possibly indicating conditionally activated or highly specialized roles. Carpel-specific expression was observed in *HvSULTR7,* showing moderate levels in CAR15 (30.45), linking it to sulfate allocation during reproductive development. Tissue-specific expression, including high levels in ROOT, LEA, and SEN, further highlights their importance in nutrient transport and redistribution during plant development and senescence.

The stress-specific expression patterns revealed substantial variability across *HvSULTR* genes in response to abiotic stress conditions such as salt, drought, waterlogging, cold, and heat (Figure 4B). Under salt stress, *HvSULTR6* exhibited high expression (65.14 in salt_control and 17.70 in salt_stress). At the same time, *HvSULTR10* showed moderate expression (5.54 in salt_control and 4.64 in salt_stress), suggesting their involvement in maintaining sulfate homeostasis during salinity. Drought stress elicited strong expression from *HvSULTR5* (76.75 in drought_control and 134.01 in drought_stress) and *HvSULTR2* (68.89 in drought_control); these genes are key players in sulfate redistribution during water deficit conditions. Waterlogging stress resulted in a high expression of *HvSULTR8* (61.37 in waterlogging_control and 71.69 in waterlogging_stress), highlighting its role in sulfate transport under excess water conditions. Similarly, *HvSULTR10* exhibited moderate expression (9.24 in waterlogging_stress), suggesting auxiliary roles in ion and water balance. Under cold stress, *HvSULTR8* and *HvSULTR2* demonstrated moderate expression in cold_control (14.21 and 31.10, respectively) and cold_stress (20.54 for both), reflecting their role in maintaining sulfate regulation during low-temperature stress. The highest heat-responsive expression was observed in *HvSULTR5* (157.90 in heat_stress), indicating its critical role in sulfate homeostasis during high-temperature conditions. Other genes, such as *HvSULTR10,* also showed moderate expression under heat stress (5.61 in heat_stress). Genes such as *HvSULTR6* and *HvSULTR5* emerged as key candidates for abiotic stress tolerance, exhibiting robust expression under multiple stress conditions. These findings provide compelling evidence of the diverse functional roles of *HvSULTR* genes in sulfate uptake, transport, and stress adaptation.

### 2.4. Response of HvSULTRs to Heat Stress in Barley Leaves

**Figure 5:**
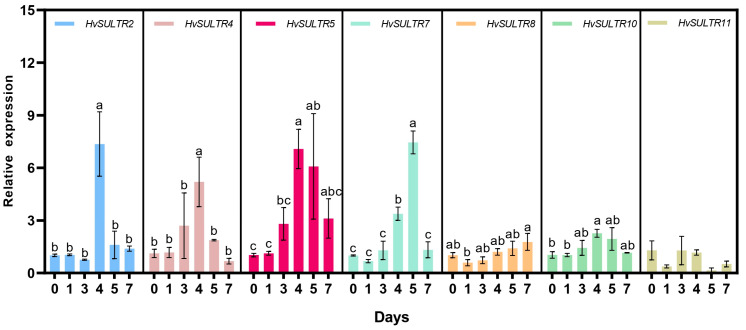
Gene expression analysis of barley leaves under control and heat treatment. The profile level of control plants (0 d) was set to 1.0. Data are means of three independent replicates ± SD. Diverse letters indicate significant differences (*p* < 0.05).

The importance of *HvSULTRs* in barley’s adaptive response to abiotic stresses was further investigated by quantifying the expression levels of seven *HvSULTR* members upon heat stress (Figure 5). Our results show that these *HvSULTRs* (except *HvSULTR11*) were all induced by heat stress. Still, they exhibited different response trends with the exposure time. For instance, *HvSULTR2*, *HvSULTR4*, *HvSULTR5*, and *HvSULTR10* reached their highest expression levels on the fourth day of heat stress and then recovered to baseline. A similar trend was observed for *HvSULTR7*, with the peak value of its expression appearing on the fifth day. Notably, under heat stress treatment, the expression level of the *HvSULTR8* gene was continuously upregulated, showing an expression pattern associated with heat stress response. Based on this gene expression-level result, it can be preliminarily inferred that such *HvSULTR* genes may be involved in regulating the adaptive response of barley to heat stress, but their specific functions require further verification in combination with protein activity assays. It was unexpected that the expression level of *HvSULTR11* showed little change under heat stress, which suggests that this gene may have a distinct role in barley’s response to heat stress.

### 2.5. Interaction of Barley HvSULTR11 with Hv14-3-3A/D and Its Subcellular Localization

**Figure 6:**
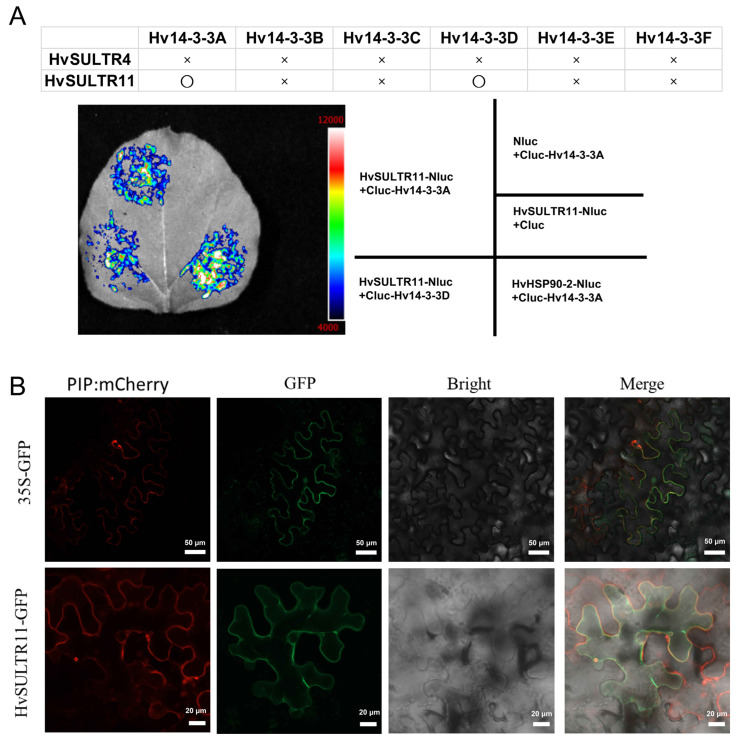
Interaction of barley HvSULTR11 with Hv14-3-3A/D and its subcellular localization. Interaction of barley HvSULTR and Hv14-3-3A/D, as revealed by the LUC assay (**A**). Summary of the interaction of SULTR11/SULTR4 with 14-3-3A/B/C/D/E/F, as revealed through the LUC assay, in tobacco leaves. O, strong interaction; ×, no interaction. Subcellular localization of HvSULTR11 (**B**), bar = 50 μm in the 35S-GFP, and bar = 20 μm in the HvSULTR11-GFP.

Hv14-3-3A positively regulates stress tolerance in barley [56]. Herein, the interaction between HvSULTR and Hv14-3-3s was examined using a luciferase complementation (LUC) assay. When Cluc-fused HvSULTR11 and Hv14-3-3A plasmids were co-expressed in tobacco leaves, a strong signal was observed, in contrast, no signal was detected in the negative control (Figure 6A). Similarly, an interaction between HvSULTR11 and Hv14-3-3D was also observed (Figure 6A). Still, no interactions were found between HvSULTR and other Hv14-3-3 proteins (Figure 6B). Additionally, no direct interaction was detected between HvSULTR4 and Hv14-3-3s (Figure 6B). Our results indicate that there are specific interactions between HvSULTR11 and Hv14-3-3A/D in barley.

The subcellular localization of HvSULTR11 was then investigated by transiently co-expressing HvSULTR11-GFP fusion construction with pNC-Green-SubN vector and plasma membrane marker PIP: mCherry on the tobacco leaves. Confocal fluorescence microscopic results show that the HvSULTR11-GFP fluorescence was merged with the red color of the marker (Figure 6B), confirming that HvSULTR11 is localized to the plasma membrane.

### 2.6. Genetic Variation in SULTR11 Gene Haplotypes and Population Structure in Cultivated, Wild, and Tibetan Barley Collections

**Figure 7:**
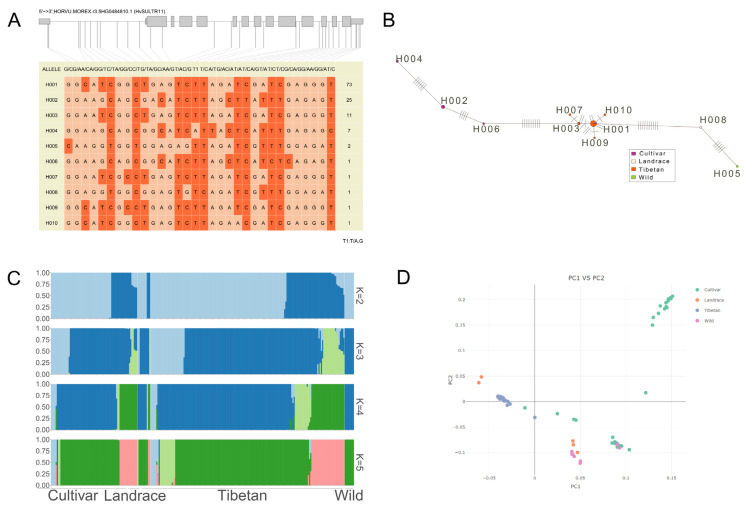
Haplotype of HvSULTR11 in cultivar, wild, landrace, and Tibetan barley. Gene haplotype in diverse barley (**A**), red representing T/C and light red representing A/G. Haplotype network relationship (**B**). Population genetic structure of different barley (**C**), the four small graphs represent population structure analysis based on different clustering numbers (K = 2 to K = 5), and the color blocks in each small image represent different genetic components or ancestral sources. Scatter plot of population genetic structure based on principal component analysis (PCA) (**D**). Data were downloaded from a public database (https://www.barleygvdb.cn/project/?pro=WGS&_rand=buu1q accessed on 8 July 2025).

In the coding region of the *SULTR11* gene, there are thirty-two SNP sites, which together form ten haplotypes. Among them, the haplotypes of the three barley collections (cultivated, wild, and Tibetan) exceed ten. Among them, the cultivated varieties were mainly concentrated on H002, H004, and H006 (Figure 7B). There were 73 numbers of H001, 25 numbers of H002, and 11 numbers of H003, which were statistically significant (Figure 7A). Through the visualization graph of the population structure analysis results (Figure 7C), when K = 2, the population was divided into wild and cultivated groups, with landraces showing transitional mixing; as K increased, the groups were further subdivided. Landraces and the Tibetan group accumulated more genetic differentiation due to selection, while cultivated varieties had a relatively concentrated genetic background due to breeding or artificial selection.

Meanwhile, principal component analysis showed that cultivated varieties form a relatively independent cluster (with consistent genetic background), wild groups are far from cultivated groups in clustering (with significant genetic differentiation), and the independent clustering of the Tibetan group provides clues for research on plateau adaptation genes (Figure 7D).

## 3. Discussion

### 3.1. Sulfate Transporter Genes Exhibited Evolutionarily Conserved Functions

The *SULTR* orthologs exist in terrestrial plants and algae, which may have originated from *Chromista* (e.g., *Pavlova lutheri* and *Prymnesium parvum*) (Figure 1A). The results suggest that SULTRs are highly conserved in higher plants and algae. In addition, the number of *SULTR* genes is lower in Chlorophyte algae. In contrast, angiosperms have a higher number of *SULTR* genes, which indicates that *SULTRs* have undergone whole-genome duplications (WGDs) in angiosperms (Figure 1B). Interestingly, the number of *SULTR* genes might increase by WGD events in *A. thaliana*, oilseed rape, rice, maize, and other species. The number of these genes may gain a new function in the process of evolution, which might be due to adapting to different environments and plant growth [17,19,57,58]. In *A. thaliana*, SUTLR proteins, based on phylogenetic relationships, can be divided into four different subfamilies, SUTLR1–SUTLR4 [6]. Consistent with studies on other species, such as soybean, potato, *Malus domestica* [59,60,61], and barley (Figure 3), different subfamilies of SULTR differ in subcellular localization, expression pattern, and substrate affinity, indicating that SULTR underwent gene duplications and eventually expanded to multiple subfamily members, playing a role in the sulfate transport process. A total of 16 *SULTR* genes were identified in barley through conserved domain alignment in this study. Meanwhile, this indicated preliminary evidence that the *SULTR* gene family in barley may have undergone gene replication and expansion by phylogenetic analysis (Figure 3). According to previous reports, there are two different evolutionary trajectories of sulfate transporter families in green algae and terrestrial plants [9,19]. Thus, *SULTRs* in barley may have various functions due to different evolutionary origins. Notably, *SULTR* has a conserved motif that may play an important role in sulfate transport and environmental adaptation.

### 3.2. Sulfate Transporter Genes Response to Abiotic Stress in Plants

SULTR are the most vital transporters in plant sulfur metabolism, which is responsible for absorbing sulfate from the environment and transporting and distributing sulfate [7]. However, a range of evidence has been indicated that *SULTR* also plays essential roles in response to abiotic stresses, such as drought, salt, and heavy metal stress [18,62,63,64,65]. The *SULTR* genes family have been identified in many plant species, such as rice [58], soybean (*Glycine max*) [60], *Malus domestica* [59], *Brachypodium distachyon* [66], and *Solanum tuberosum* [61].

For instance, the seed germination rate of the *AtSULTR3* quintuple mutant was significantly lower than that of WT under ABA or high salt stress [67]. Disruption of *AtSULTR1;1* and *AtSULTR1;2* reduced glutathione content and led to increased sensitivity to cadmium (Cd) [68]. In addition, sulfate has been presumed to be an early signal that plants are responding to drought stress [69]. Therefore, *SULTRs* were also indirectly involved in drought stress. Recently, ancient hybridization events significantly promoted gene duplication and evolutionary rate shifts in the *SULTR* family during the early evolution of *Caragana arborescens*, which revealed that *SULTR* gene was closely related to drought adaptation [70]. Overexpression of *OsSultr1;1* in *A. thaliana* improved growth under limiting sulfur and show tolerance towards arsenic and abiotic stress [22]. OsSULTR3.4, HvSULTR3.4, and AtSULTR3.4 are all internal transport carriers of phosphate, and their knockout mutants show impaired phosphorus distribution in developing organs [55,71,72], while PtaSULTR3.4a participates in the phosphate starvation response and is also strongly co-expressed with lignification and one-carbon metabolism genes and their upstream transcription regulators [73]. Therefore, SULTRs play important roles in plant response to abiotic stresses and biological processes. So far, the functional verification on *SULTR* genes under abiotic stress remains limited. Hence, most studies mainly focus on the expression patterns of *SULTRs* under diverse stress conditions. Recent studies show transcript changes in most of the *SULTR* genes in cotton (*Gossypium genus*), maize (*Z. mays*), and rice in response to salt, drought, and heat stress [14,57,58]. In our study, the expression patterns of most *HvSULTR* genes in response to heat stress were first significantly up-regulated and then down-regulated (Figure 4 and Figure 6), which was consistent with transcriptomic data. The transcriptomic data also displayed a remarkable expression change in *HvSULTRs* under other abiotic stress, especially *HvSULTR2* and *HvSULTR5*. The expression level of *HvSULTR5* in shoot strongly increased under heavy metal stress (Figure S3). Interestingly, the transcripts of *HvSUTLR11* have no significant change in GP. However, there are four main haplotypes of *HvSUTLR11* (Figure 7), which suggests that natural variation in *HvSULTR11* might change barley heat tolerance in different varieties. Thus, the barley *SULTR* genes might play crucial roles in abiotic stress. However, the specific role of *HvSULTR* genes in abiotic stress tolerance should be further investigated by constructing barley mutant lines and overexpression lines.

### 3.3. Regulation of SULTR Proteins by Their Interacting Proteins

Although SULTR is crucial for the transport and absorption of sulfates in plants, little is known about SULTR transcriptional regulation. It has been reported that Sulfur Limitation1 (SLIM1) may have dual functions as an activator during sulfur limitation and as a repressor during normal sulfur states and influencing the expression of downstream *SULTR* genes [74,75]. Further research reported that SLIM1 induces microRNA395, specifically targeting ATP sulfurylases and a low-affinity sulfate transporter, SULTR2;1, thus affecting sulfate assimilation and transport in *A. thaliana* [76]. LUC assay showed that HvSULTR11 interacts with Hv14-3-3A and Hv14-3-3D, which is similar to a previous study [77]. 14-3-3 proteins mainly interact with target proteins by recognizing the phosphorylation sites on them, leading to significant changes in the stability, subcellular localization, or interaction with other proteins of the target proteins, and thereby regulating the functions of the target proteins. They also play pivotal roles in the growth, development, and abiotic stress in plants [78,79,80]. In *Malus domestica*, the 14-3-3 protein General Regulatory Factor 8 (GRF8) binds to the phosphorylated form of WRKY18, enhancing its stability and transcriptional activation activity. Then, MdWRKY18 activates Salt-Overly-Sensitive 2 (SOS2) and SOS3 in response to salt stress [80]. MdGRF11 promotes the degradation of the nitrate-responsive BTB/TAZ (BT2) protein, thereby increasing the abundance of MdMYB1 protein, which in turn induces anthocyanin accumulation in response to nitrate deficiency [81]. The OsGF14f protein interacts with Basic (region) Leucine Zipper 23 (bZIP23), enhancing its transcriptional regulatory function and activating the expression of downstream stress response genes and increasing tolerance to osmotic stress [82]. In the response to cold stress of *A. thaliana*, Cold Responsive Protein Kinase 1 (CRPK1) phosphorylates 14-3-3 protein, promoting its entry into the nucleus to bind with C-repeat binding factor (CBF) and facilitating the degradation of CBF, thereby affecting the expression of cold stress-related genes [83]. In this study, we found that HvSULTR11 interacts with Hv14-3-3A and Hv14-3-3D. Unexpectedly, the relative expression level of *HvSULTR11* have no significant change under heat stress. However, 14-3-3 proteins play vital roles in heat, drought, salt, cold, and osmtic stress [78,84]. Therefore, we proposed that Hv14-3-3s may regulate sulfur transport or respond to abiotic stress by phosphorylating HvSULTR11, which might affect the protein expression level rather than the transcriptional level. In addition, whether there are other proteins or transcription factors involved in the 14-3-3s–SULTRs module that respond to abiotic stress also needs further investigation.

## 4. Materials and Methods

### 4.1. Identification, Nomenclature, Phylogenetic, and Structure Analysis of Sulfate Transporter Genes

To identify the Solfataras (PF00916) [66] and STAS (PF01740) [61] proteins in *H. vulgare*, a Hidden Markov Model (HMM) profile was conducted to identify the putative proteins from genome sequences using the software HMMER 3.4.0.2 [85] with a cut-off E-value of < 1 × 10^−20^ Names were given to *SULTR* genes through the location on the respective chromosome according to a previous study [86]. The phylogenetic tree was conducted using MEGA 7.0 software, and the results are displayed using iTOL 7.0 [87].

The conserved motifs and regions of SULTR proteins were identified using the MEME tool. And the maximum number of motifs was set to 10 and the optimum motif width was ≥6 and ≤50 [56]. TBtools was used to visualize both the gene structure and motif composition [88].

### 4.2. Expression Analysis of SULTR in Various Tissues and Abiotic Stress

To create the expression profile of *SULTR* genes among diverse organs and development stages, the RNA-seq data from different tissues in barley were downloaded from IPK (https://apex.ipk-gatersleben.de/apex/f?p=284:49 accessed on 9 July 2025). The development stages include roots from the seedlings (10 cm shoot stage) (ROO1), shoots from the seedlings (10 cm shoot stage) (LEA), young developing inflorescences (5 mm) (INF1), developing inflorescences (1–1.5 cm) (INF2), developing tillers, 3rd internode (NOD), developing grain (5 DAP) (CAR5), developing grain (15 DAP) (CAR15), etiolated seedling, dark condition (10 DAP) (ETI), inflorescences, lemma (42 DAP) (LEM), inflorescences, lodicule (42 DAP) (LOD), epidermal strips (28 DAP) (EPI), inflorescences, rachis (35 DAP) (RAC), Roots (28 DAP) (ROO2), and senescing leaves (56 DAP) (SEN). The transcript abundance of anion channel genes was calculated according to [89].

Raw expression values of *SULTR* genes were retrieved from the CoNekT database [90]. These species include *A. thaliana*, *O. sativa*, *Z. mays*, *S. lycopersicum*, *A. trichopoda*, *P. abies*, *G. biloba*, *S. moellendorffii*, *P. patens*, and *M. polymorpha*. Sampling conditions were categorized into apical meristem, root meristem, seeds, flower, stem, leaf, female (ovaries, pistils), and male (pollen, anthers).

We analyzed the public transcriptomics data for the roles of SULTRs in abiotic stresses using *A. thaliana*, barley, and *P. patens*. Barley *HvSULTRs* datasets were obtained from various stress treatments such as drought [91], submergence [92], high temperature [93], cold [94], and salinity [95]. The expression of *AtSULTRs* was obtained from studies of drought [96], low temperature [97], heat stress [98], waterlogging [99], and salt [100] stresses. The expression of *SULTRs* in *P. patens* was investigated via studies of drought, cold, salt [101], and heat [102] treatments. Data were downloaded from a public database (http://barleyexp.com/) [103].

### 4.3. Firefly Luciferase Complementation (LUC) Imaging and Subcellular Localization Assay

The LUC assay was used to perform protein–protein interactions. The coding DNA sequence (CDS) of *Hv14-3-3s* and *HvSULTRs* were introduced into pCAMBIA2300-CLuc and pCAMBIA2300-NLuc, respectively [104,105]. The resulting colonies containing the expression plasmids were grown in LB medium (50 µg/mL Kanamycin and 25 µg/mL Rifampicin) overnight. After centrifugation and resuspending, they were infiltrated into 4-week-old *Nicotiana benthamiana* leaves. After 3 d, the leaves of tobacco were sprayed with D-luciferin potassium salt substrate (1 mM, Macklin) and kept in dark conditions for 10 min [56]. The LUC signals were imaged using a Tanon 5200 chemiluminescence imaging system (Tanon Science, Shanghai, China).

Subcellular localization of HvSULTR11 was conducted according to our study [106]. The coding regions of HvSULTR11 were amplified and cloned into pNC-Green-SubN by the restriction enzyme site *SfiI*. The resulting plasmids were transferred into the Agrobacterium strain GV3101. Agrobacterium harboring the vector was grown overnight in Luria-Broth (LB) medium containing 25 mg/L of Rifampin and 50 mg/L of Kanamycin [56]. After centrifugation, Agrobacterium was resuspended in the infiltration buffer [10 mM 2-(N-morpholino) ethanesulfonic acid (MES)-KOH (pH 5.7), 10 mM MgCl2, 100 μM acetosyringone (AS)] to achieve OD600 = 0.8. The suspension was infiltrated into the abaxial air spaces of 4-week-old N. benthamiana leaves using a 1 mL syringe without a needle to express transiently [51]. GFP fluorescence was detected by using confocal microscopy (Zeiss LSM900, ZEISS, Oberkochen, Germany). Confocal imaging settings were excitation at 488 nm and emission at 500–530 nm (GFP) and 600–660 nm (chloroplast) [107].

### 4.4. qPCR Analysis of Sulfate Transporters Under Heat Treatment

Seven differentially expressed sulfate transporters were selected to confirm their response in barley plants under heat conditions. Total RNA was extracted from leaves after heat treatment (0, 1, 3, 4, 6, and 7 days) from the plants using an RNA extraction kit (Aid lab, Beijing, China, RN38-EASYspin). The qScript cDNA Synthesis Kit (Takara, Shiga, Japan) was conducted for cDNA synthesis, and the synthesized cDNA was diluted 5 times for qPCR [56]. The qPCR was performed using SYBR green PCR master mix (ABI) and the Light Cycler 96 Real-Time PCR System (CFX Connect, Bio-Rad, Hercules, CA, USA) with three biological replicates. The expression levels were normalized against the *HvActin* reference gene. The gene primers of qPCR are displayed in Table S1. The relative expression levels of *SULTR* genes were determined from cycle threshold values by the 2^−ΔΔCt^ procedure [85].

### 4.5. Statistical Analysis

Data are shown as means with standard errors of three independent biological replicates. SPSS 26.0 software (IBM, USA) was employed to perform the analysis of variance (ANOVA), and means were compared using Duncan’s multiple range tests.

## Data Availability

The original contributions presented in this study are included in the article/Supplementary Materials. Further inquiries can be directed to the corresponding author.

## Supplementary Materials

The following supporting information can be downloaded at: https://www.mdpi.com/article/10.3390/plants14203165/s1.
