# Supplementary material for: Molecular Evolution of Plant SULTR Proteins and Expression Analysis of HvSULTR Under Heat Stress in Barley
Source: Plants (Basel). 2025 Oct 15;14(20):3165. doi: 10.3390/plants14203165 (PMC12567104; doi:10.3390/plants14203165)
Supplement: Supplementary file 1 [file plants-14-03165-s001.zip › plants-3811027-supplementary.pdf]

Table S1. **List of primer sequences used in this study.**

Primer name	Primer Sequence
Sultr2-qPCR-F	GGATCTATCAGCTGTCCCTGC
Sultr2-qPCR-R	GTGTGCATCGTTCGCTCG
Sultr4-qPCR-F	GAGGAGTCCGAGAAGACAGAAC
Sultr4-qPCR-R	GCCTTCTCGTCCGCGTTAAG
Sultr5-qPCR-F	CGCGAGAGGAGGAGAAGAGT
Sultr5-qPCR-R	GGATTTGATGGACTCCGCG
Sultr7-qPCR-F	TCAGGCAGTACCCTGTGG
Sultr7-qPCR-R	CCACGTTTGACATGTCAAGGA
Sultr8-qPCR-F	GAGACCGGCGTGCAGTAC
Sultr8-qPCR-R	GGGAAGATCCACTCGTGGC
Sultr10-qPCR-F	GCGACATCCAGATCGCTATAG
Sultr10-qPCR-R	CATTCGACGATGAACTCTGCAC
Sultr11-qPCR-F	AAGAAGACAGAGCTAAAGCAGT
Sultr11-qPCR-R	AGCTTCGATGCTTGCAGTTTTT
Y-SULTR11-F	agtggtctctgtccagtcctATGGTTCATCATATATCTGACGAGGC
Y-SULTR11-R	ggtctcagcagaccacaagtTCACGGTTCCTGCATCGACTTC

Table S2.**SULTR gene nomenclature**

Gene ID	Designation
HORVU.MOREX.r2.2HG0113450.1	*HvSULTR1*
HORVU.MOREX.r2.2HG0169630.1	*HvSULTR2*
HORVU.MOREX.r2.3HG0236740.1	*HvSULTR3*
HORVU.MOREX.r2.4HG0293270.1	*HvSULTR4*
HORVU.MOREX.r2.4HG0293280.1	*HvSULTR5*
HORVU.MOREX.r2.4HG0332690.1	*HvSULTR6*
HORVU.MOREX.r2.4HG0332740.1	*HvSULTR7*
HORVU.MOREX.r2.4HG0334620.1	*HvSULTR8*
HORVU.MOREX.r2.5HG0351940.1	*HvSULTR9*
HORVU.MOREX.r2.5HG0388480.1	*HvSULTR10*
HORVU.MOREX.r2.5HG0401430.1	*HvSULTR11*
HORVU.MOREX.r2.6HG0507520.1	*HvSULTR12*
HORVU.MOREX.r2.7HG0538640.1	*HvSULTR13*
HORVU.MOREX.r2.7HG0579730.1	*HvSULTR14*
HORVU.MOREX.r2.7HG0606090.1	*HvSULTR15*
HORVU.MOREX.r2.7HG0614570.1	*HvSULTR16*

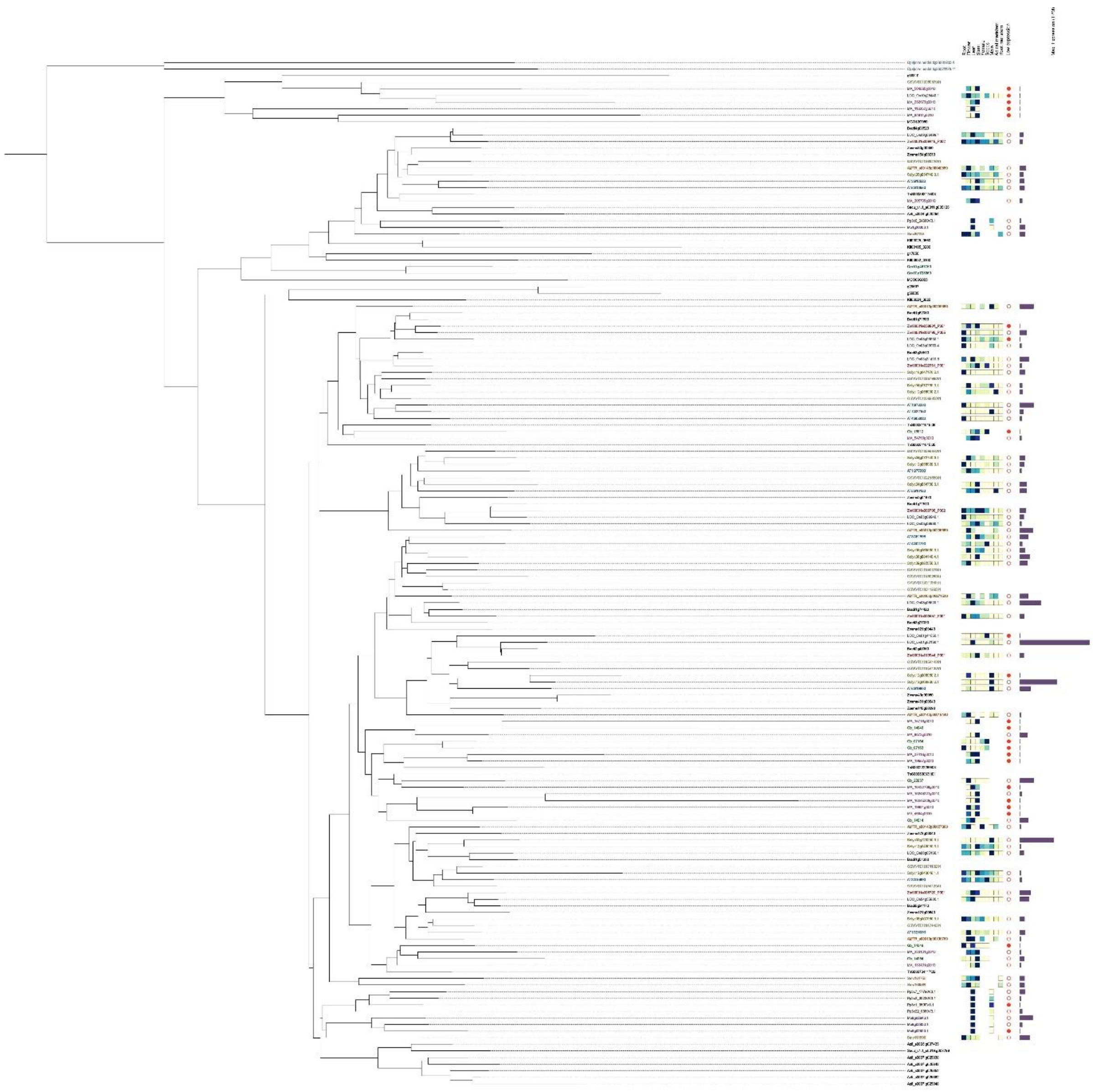

Figure S1. Phylogenetic analysis and expression patterns of *SULTR* genes in green plants. Data were downloaded from public database (https://evorepro.sbs.ntu.edu.sg/heatmap/comparative/family/283/raw, https://evorepro.sbs.ntu.edu.sg/heatmap/comparative/family/2402/raw).

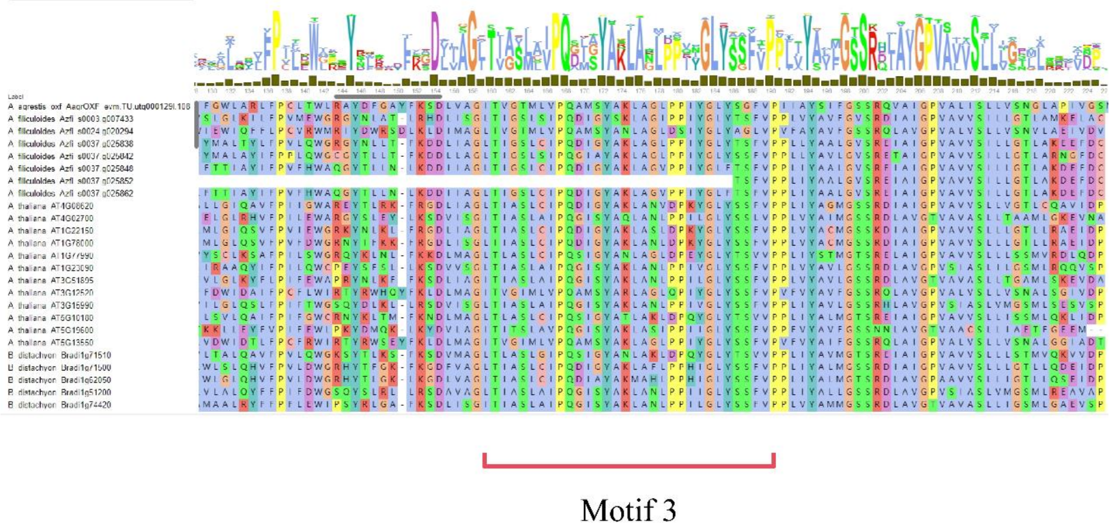

Figure S2. Motif of SULTR in multiple species. Data were downloaded from public database (https://rshiny.gwdg.de/apps/streptonet/).

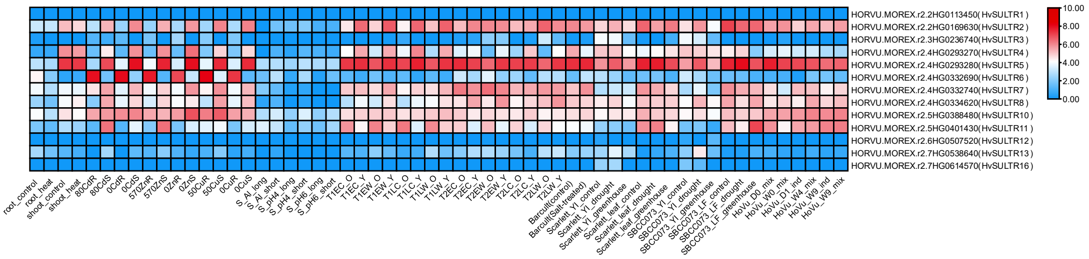

Figure S3. Expression levels of *HvSULTR* under multiple stresses. Data were downloaded from public database (http://barleyexp.com/).
